# Knowledge, Attitude, and Associated Factors towards Nonpharmacological Pain Management among Nurses Working at Amhara Region Comprehensive Specialized Hospitals, Ethiopia

**DOI:** 10.1155/2021/7374915

**Published:** 2021-11-30

**Authors:** Esayas Alemshet Tekletsadik, Abebaw Alemayehu Desta, Belayneh Shetie Workneh

**Affiliations:** ^1^Department of Surgical Nursing, School of Nursing, College of Medicine and Health Sciences, University of Gondar, Gondar, Ethiopia; ^2^Department of Emergency and Critical Care Nursing, School of Nursing, College of Medicine and Health Sciences, University of Gondar, Gondar, Ethiopia

## Abstract

**Background:**

Nonpharmacological pain management refers to providing pain management intervention that does not involve the use of drugs. Effective management of a patient's pain is a vital nursing activity, and it needs a nurse's adequate pain-related knowledge and a favorable attitude. Globally, many studies stated that the lack of knowledge and unfavorable attitude of nurses towards nonpharmacological pain management was the prevailing persistent challenge.

**Objective:**

To assess knowledge, attitude, and associated factors towards nonpharmacological pain management among nurses working in Amhara region Comprehensive Specialized Hospitals, Ethiopia, 2021.

**Method:**

An institutional-based cross-sectional study was conducted from April to May 30, 2021. A total of 845 nurses were selected using a simple random sampling technique. Data were collected by using a pretested self-administered structured questionnaire. Then, data were checked, coded, and entered into Epi Info version 7.0 and exported to SPSS version 25.0 software for analysis.

**Results:**

A total of 775 nurses participated in this study, with a response rate of 91.8%. Of the total participants, 54.2% (95% CI: 50.6–57.9) and 49.8% (95% CI: 46.1–53.2) of nurses had adequate knowledge and a favorable attitude, respectively. The study revealed that educational status (AOR = 3.51 (95% CI: 1.37, 8.99)), years of experience (AOR = 5.59 (95% CI: 2.86, 10.94)), working unit (AOR = 5.61 (95% CI: 2.25, 13.96)), nurse-to-patient ratio (AOR = 2.33 (95% CI: 1.44, 3.78)), and working hours (AOR = 2.15 (95% CI: 1.27, 3.62)) were significantly associated. This finding also revealed that monthly income (AOR = 4.38 (95% CI: 1.64, 11.69)), nurse-to-patient ratio (AOR = 1.89 (95% CI: 1.19, 3.01)), and nurses' adequate knowledge (AOR = 4.26 (95% CI: 2.91, 6.24)) were significantly associated with the attitude of nurses. *Conclusion and Recommendations*. More than half and nearly half of the nurses had adequate knowledge and a favorable attitude towards nonpharmacological pain management, respectively. Educational qualification, years of experience, working unit, nurse-to-patient ratio, and prolonged working hours per day were significantly associated with nurse's adequate knowledge. Monthly income, nurse-to-patient ratio, and nurse's knowledge were significantly associated with the attitudes of nurses. It is better to give attention to reviewing the nursing curriculum, achieving a standardized nurse-to-patient ratio, recruiting additional nurses, training, and upgrading nurses with continuing education.

## 1. Introduction

Pain is defined as “an unpleasant sensory and emotional experience associated with actual or potential tissue damage” [1, 2]. Nonpharmacological pain management (NPPM) refers to the utilization of pain management alternatives other than pharmacological therapies such as physical therapy, occupational therapy, comfort therapy, psychosocial therapy, or counseling for the better management and reduction of pain [3, 4].

Globally, many studies stated that lack of knowledge and unfavorable attitude of nurses towards NPPM was the prevailing persistent challenge [5]. Effective management of a patient's pain is a vital nursing activity, and it needs a nurse's adequate pain-related knowledge and a favorable attitude [6]. Nurse's knowledge and attitude have a significant effect on the use of NPPM. Different studies show that, among nurses who have inadequate knowledge and an unfavorable attitude towards NPPM, the majority of them did not use NPPM [7]. Moreover, among previous studies conducted regarding NPPM, 90% of them had no documented evidence of the use of nonpharmacological pain interventions to alleviate pain [8].

This enormous gap negatively affects a collective of hospitalized patients' physical, emotional, and spiritual well-being, alters the quality of life of patients, increases the incidence and severity of complications, and increases healthcare costs [9]. Ineffectively managed pain affects the patient's quality of life negatively, which results in higher hospital readmission rate, more repeated outpatient visits, prolonged hospital stay, increased risk of nosocomial infection, and also increased stress and anxiety for the patient as well as his family [10, 11].

Most of the patients who lived with pain use opioids and long-term use of these drugs have an impact on the life of the patient; they can cause dependence, impaired memory, drowsiness, tolerance, lack of concentration, and poor judgment [12]. Therefore, NPPM strategies have more advantages for managing mild to severe pain than pharmacological pain management because NPPM is cost-effective, easy to provide for patients, has high potential to relieve the patient's pain, and can be used in combination with drugs or alone [13]. According to previous studies, nurses can acquire additional knowledge of NPPM through work experience, on-the-job training, and interaction with colleagues [14].

CDC recommends nonpharmacological interventions as a first-line approach for the treatment of mild to moderate pain, but opioids are the most commonly used currently [15]. Previously, few healthcare institutions in Ethiopia tried to implement important NPPM methods [8]. However, little was known about nurses' knowledge and attitude towards NPPM. Most of the studies conducted before included a single institution and working unit, while pain management is the concern of multiple institutions and working units. Therefore, the main aim of this study was to assess nurses' knowledge, attitudes, and factors associated with nonpharmacological pain management among nurses working in Amhara region Comprehensive Specialized Hospitals in 2021.

## 2. Methods

### 2.1. Study Design

An institutional-based cross-sectional study design was employed.

### 2.2. Study Area and Period

The study was conducted from March to April 30, 2021, at Comprehensive Specialized Hospitals in the Amhara regional state. According to the Amhara National Regional Health Bureau's Annual Performance Report, the region has 81 hospitals, 858 health centers, and 3560 health posts. Among those 81 hospitals in the region, the University of Gondar, Dessie, Felege-Hiwot, Tibebe-Ghion, Debre Markos, Woldia, Debre Tabor, and Debreberhan are Comprehensive Specialized Hospitals. At the time of conducting this study, 1985 nurses were working in those hospitals. Thus, all those eight comprehensive specialized hospitals serve the population found in the region [16].

### 2.3. Population

All nurses working in Amhara regional state Comprehensive Specialized Hospitals were the source population. All nurses who were working in selected Comprehensive Specialized Hospitals during the data collection period were the study population.

### 2.4. Eligibility Criteria

#### 2.4.1. Inclusion Criteria

All nurses who were working in selected Comprehensive Specialized Hospitals during the data collection period were included.

#### 2.4.2. Exclusion Criteria

Those nurses who were seriously ill (unable to respond) were excluded from the study.

### 2.5. Sample Size Determination

The sample size was determined by using the single population proportion formula by taking the proportion of knowledge (51.2%) and the favorable attitude (47%) [8], 95% confidence interval, and 5% marginal error.

(1) $$\begin{array}{c} n=\frac{{\left(Za/2\right)}^{2}*P\left(1-P\right)}{d^{2}}, \end{array}$$

where *n* is the required sample size, *Za*/2 is the standard normal deviation at 95% CI, *P* is the proportion of knowledge (51.2%), *d* is margin of error that can be tolerated (5%), and 1 − *p* is the proportion of the population that does not possess the character of interest.

Therefore,

(2) $$\begin{array}{c} n=\frac{{\left(Za/2\right)}^{2}*P\left(1-P\right)}{d^{2}}=\frac{{\left(1.96\right)}^{2}*0.512\left(1-0.512\right)}{{\left(0.05\right)}^{2}}=384, \end{array}$$

whereas the calculated sample size for the second dependent variable (attitude) was 383.

To obtain a maximum sample size, a large number from the computed sample sizes were taken. Therefore, from the calculated sample sizes, the largest was 384. Since the sampling technique was multistage simple random sampling, the design effect was considered. So the sample size was multiplied by the number of stages (i.e., 2) and gives 768. The final sample size was 845 after using the design effect and adding a 10% nonresponse rate.

### 2.6. Sampling Technique and Procedure

**Figure 1:**
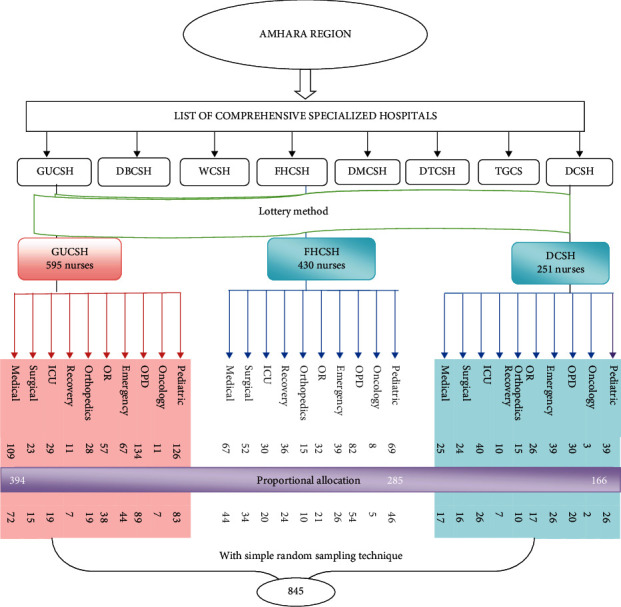
Schematic presentation of sampling procedure on knowledge, attitude, and associated factors of nurses towards non-pharmacological pain management among nurses working at Amhara region Comprehensive Specialized Hospitals, Ethiopia, 2021.

A simple random sampling technique was used to select the study participants. Among the eight government Comprehensive Specialized Hospitals which are found in the Amhara region (Gondar University Comprehensive Specialized Hospital, Debre Markos Comprehensive Specialized Hospital, Felege-Hiwot Comprehensive Specialized Hospital, Debreberhan Comprehensive Specialized Hospital, Tibebe-Ghion Comprehensive Specialized Hospital, Debre Tabor Comprehensive Specialized Hospital, Woldia Comprehensive Specialized Hospital, and Dessie Comprehensive Specialized Hospital), three Comprehensive Specialized Hospitals (Gondar University Comprehensive Specialized Hospital, Dessie Comprehensive Specialized Hospital, and Felege-Hiwot Comprehensive Specialized Hospital) were selected with lottery method. Then, the total sample size was proportionally allocated for each working unit of the selected hospitals in which nurses are working (Figure 1).

### 2.7. Operational Definitions

  Adequate knowledge: nurses who had scored median and above on the knowledge-related questions were considered as having adequate knowledge
  Inadequate knowledge: those nurses who scored below the median on the knowledge questions were considered as having inadequate knowledge
  Favorable attitude: nurses who had scored median and above on the attitude-related questions were considered as having a positive attitude
  Unfavorable attitude: nurses who had scored below the median on the attitude-related questions were considered as having a negative attitude

### 2.8. Data Collection Instruments and Procedures

A structured self-administered questionnaire (NKASRP) adapted from other similar studies was used to collect the data [8, 17–19]. The questionnaires were prepared in English in the form of closed-ended questions. The questionnaires had four sections. The 1^st^ section was composed of 12 sociodemographic characteristics of participants including age, sex, educational status, year of experience, and formal training regarding nonpharmacological pain management. The 2^nd^ section was composed of 20 questions that assessed the knowledge of participants. The 3^rd^ section was composed of 18 questions with a Likert scale which assessed the attitude of nurses, and the 4^th^ section was composed of 19 facility-related questions.

Seven clinical nurses for the data collection and three BSc nurses for supervisors were recruited. One-day training for data collectors and supervisors was delivered by the principal investigator before the beginning of data collection regarding the objectives of the study, the data collection approach, the contents and relevance of the study, the confidentiality of the information, as well as the rights of participants. Simple random sampling method was used to get participants. Then, after eight hundred forty-five questionnaires were distributed, eight hundred nineteen were returned. During data collection, data collectors and supervisors followed the recommended precautions to prevent COVID-19.

### 2.9. Data Processing and Analysis

Data clean-up and checking were done before the analysis. Checked, cleaned, and coded data were entered into Epi Info version 7.0 and exported to SPSS version 25 for analysis. Reverse coding was done for negative questions to get consistent effects of measurement items. Descriptive statistics like frequencies, percentages, and tables were used to present the data. First, bivariable relationships between each independent variable and the dependent variable were investigated using a binary logistic regression model. Independent variables with a *p* value <0.25 were included in multivariable analysis to control potential confounding factors. Finally, those variables with a *p* value <0.05 with a 95% confidence interval were regarded as factors significantly associated.

### 2.10. Data Quality Assurance

The self-administered questionnaire was pretested by taking 5% of the sample size before the actual data collection time at Debre Tabor Comprehensive Specialized Hospital. After the pretest was conducted, amendments on the instrument, such as unclear questions and ambiguous words, were checked accordingly. Data collectors and supervisors were recruited, and one-day training was given on the objective of the study, instrument, and data collection procedures by the principal investigator.

Supervision was conducted by the principal investigator and supervisors. To ensure data quality, each data collector checked the questionnaire from each study participant for completeness on a daily basis. The supervisors and principal investigator reviewed each questionnaire daily and checked for completeness. Cronbach's alpha value was done to check its reliability, and the knowledge item scored 0.751 and the attitude item scored 0.882.

## 3. Results

### 3.1. Sociodemographic Characteristics of the Respondents

A total of 775 nurses participated in this study, with a 91.8% response rate. More than half (57.2%) of the respondents were females, and 55.4% of them were married. The median age of study participants was 30 years with an IQR of 28–35, and the minimum and maximum ages of respondents were 23 and 57, respectively, and three hundred forty-six (44.6%) of the study participants fall into the age category between 30 and 39 years. Concerning the monthly salary, three hundred forty-two (44.1%) of participants earned a monthly income of <5358 Ethiopian birr (Table 1).

### 3.2. Professional and Organizational Related Factors

Out of the total respondents, 299 (38.6%) reported that the nurse-to-patient ratio in their working unit was undetermined, while 236 (30.5%) reported a nurse-to-patient ratio of 1 : 4. Of the total participants, about half (395, 51%) reported that there was no pain assessment tool in their working units. Nearly two-thirds (586, 75.6%) of the study participants had no previous training on nonpharmacological pain management. Of the total participants, 546 (70.5%) reported the presence of workload in their working unit, and 128 (16.5%) worked more than 12 hours per day. Among the participants, four hundred sixty-two (462, 59.6%) were degree holders, and eighty-eight (88, 11.4%) were master's degree holders. Of the total participants, 53.5% of the respondents were attending courses on nonpharmacological pain management, and about 78.5% of the study participants reported that they had previously used this kind of pain management method (Table 2).

### 3.3. Knowledge of Nurses regarding Nonpharmacological Pain Management

Out of the twenty knowledge test questions distributed to the respondents regarding nonpharmacological pain management, 54.2% of nurses (95% CI: 50.6, 57.9) had adequate knowledge regarding nonpharmacological pain management with a total median knowledge score of 70%. Out of the total twenty knowledge-related questions, the minimum score was 4 and the maximum score was 20 with a median score of 14 (IQR, 12–18) (Table 3).

### 3.4. The Attitude of Nurses towards Nonpharmacological Pain Management

Of the total of 775 participants, this study showed that 49.8% (95% CI: 46.1, 53.2) of the study participants had a favorable attitude regarding nonpharmacological pain management with a total median attitude score of 70%. Of the eighteen Likert scale attitude-related questions, the minimum score was 37 and the maximum score was 90 with a median attitude score of 63 (IQR, 56–79). Among the total participants, 379 (48.9%) strongly agreed with the idea that nurses are the best judges of the patient's pain intensity than doctors because they spend 24 hours with the patient, and 253 (32.6%) agreed with the idea of nurses' willingness to provide nonpharmacological methods of pain management to patients who have pain (Table 4).

### 3.5. Factors Associated with Nurses' Knowledge

In bivariable logistic regression analysis, age, monthly income, educational status, year of experience, previous education on nonpharmacological pain management, working unit, nurse-to-patient ratio, workload, lack of evidence, training, and working hours were associated with knowledge. In multivariable logistic regression analysis, educational status, year of experience, working unit, nurse-to-patient ratio, and working hours were found to be significantly associated.

MScholders were nearly four times more likely (AOR = 3.51 (1.37–8.99)) to have adequate knowledge as compared with diploma nurses, and BSc nurses were nearly three times more likely (AOR = 2.86 (1.80–4.56)) to have adequate knowledge as compared to diploma nurses. Nurses who work in an emergency department were five (AOR = 5.10 (2.57, 10.11)) times more likely to have adequate knowledge as compared to those who work in the pediatrics unit. Nurses who reported a nurse-to-patient ratio of 1 : 4 in their working unit were two times more likely (AOR = 2.33 (1.44, 3.78) to have adequate knowledge compared with those who reported an undetermined nurse-to-patient ratio in their working units. Whereas nurses who reported a working hour of 8 hours per day (AOR = 2.15 (1.27, 3.62)) and who reported a working hour of 8–12 hours per day were two times (AOR = 2.09 (1.18, 3.70)) more likely to have adequate knowledge as compared to nurses who reported a working hour of >12 hours per day.

Moreover, nurses who have years of experience of >5 years were nearly six times more likely (AOR = 5.59 (2.86–10.94)), those who have years of experience of 3–5 years were nearly four times more likely (AOR = 3.99 (2.05–7.79)), and those who have years of experience of 1–3 years were two times more likely (AOR = 2.19 (1.20–4.00)) to have adequate knowledge as compared to those nurses who reported years of experience of less than one year (Table 5).

### 3.6. Factors Associated with Nurses' Attitude

In bivariable logistic regression analysis, age, monthly income, educational qualification, year of experience, previous education on nonpharmacological pain management, working unit, nurse-to-patient ratio, workload, lack of evidence, working hours, and adequate knowledge were significantly associated with attitude. In multivariable analysis, only monthly income, nurse-to-patient ratio, and knowledge were significantly associated.

Those nurses who have a monthly income greater than 8017 Ethiopian birr were four times more likely (AOR = 4.38 (1.64, 11.69)), nurses who have a monthly income of 7071–8017 Ethiopian birr were five times more likely (AOR = 5.36 (2.78, 10.33)), and nurses who have a monthly income of 6193–7071 Ethiopian birr were three times more likely (AOR = 3.19 (1.65, 6.16)) to have favorable attitude compared with those nurses who had monthly income less than 5346 Ethiopian birr.

Moreover, those nurses who reported a nurse-to-patient ratio of 1 : 4 in their working unit were two times more likely (AOR = 1.89 (1.19, 3.01)) to have a positive attitude as compared to those who reported an undetermined nurse-to-patient ratio in their working unit. Furthermore, nurses who have adequate knowledge were four times more likely (AOR = 4.26 (2.91, 6.24)) to have a favorable attitude (Table 6).

## 4. Discussion

This study was conducted to assess nurses' knowledge, attitude, and associated factors towards nonpharmacological pain management. The study revealed that 54.2% (95% CI: 50.6, 57.9) of nurses had adequate knowledge regarding nonpharmacological pain management with a total median knowledge score of 14. This finding is consistent with studies conducted in Benishangul-Gumuz (51.2%) [8] and Addis Ababa Black Lion Hospital (52%) [20].

The result of this study is lower than a study conducted in Saudi Arabia (87.5%) [17]. The variation might be due to the differences in nursing curriculum regarding nonpharmacological pain management, sources of information, continuous and sustainable on-the-job training programs, and variations in technological advancement in the two countries.

But the result of this study is higher than a study conducted in Zimbabwe where 48.6% of the study participants had adequate knowledge [21]. The difference might be due to the variation in educational status. In a study conducted in Zimbabwe, about 58.7% of the study participants were diploma holders, but in this study, more than half (59.6%) of the study participants are degree holders, whereas diploma holders are only 29% of the total participants.

Regarding attitude, this study revealed that 49.8% of nurses (95% CI: 46.1, 53.2) had a favorable attitude towards nonpharmacological pain management with a total median attitude score of 70%. The result of this study is in line with a study conducted in Benishangul-Gumuz, Ethiopia (47%) [8].

The finding of this study is lower compared with a study conducted in Saudi Arabia (85%) [17]. These variations may be due to differences in perceptions of the study participants towards nonpharmacological pain management. Moreover, the possible justification might be due to the difference in the cutoff point of the scores regarding the attitude-related tool. That is, in this study, the cutoff point for a favorable attitude was ≥ 63, whereas the cutoff point for a favorable attitude in Saudi Arabia was >60 [17].

This finding is higher as compared with a study conducted in Addis Ababa Black Lion Hospital which revealed that 34.6% of the study participants had a favorable attitude towards nonpharmacological pain management [20]. The possible justification for this could be due to a difference in sample size (i.e., the sample size for this study is 845 whereas the sample size for the study done in Addis Ababa was 269).

Educational status, year of experience, working unit, nurse-to-patient ratio, and working hours per day were the factors significantly associated with knowledge.

Nurses who had master's degrees were nearly four times more likely, and BSc nurses were about three times more likely to have adequate knowledge as compared to diploma nurses. This is similar to studies conducted in Benishangul-Gumuz [8], Eritrea [22], Saudi Arabia [17], China [23], and the USA [24]. The possible justification for this could be that as nurses' educational status increases, they could have a higher probability of getting up-to-date information regarding nonpharmacological pain management, as well as they might have a higher chance to review different kinds of literature regarding the topic and are more likely to apply their knowledge in practice to treat the patient's pain.

This finding is supported by studies conducted in Turkey, Iran, and Norway that shows that nurses who were degree bachelorette and above had more theoretical and clinical knowledge regarding pain and its management than nondegree bachelorette nurses. Furthermore, they had more experience in clinical practice [25–27].

Nurses with experience of >5 years, 3–5 years, and 1–3 years were six times, four times, and two times more likely to have adequate knowledge, respectively, compared with nurses who have less than 1 year of experience. This study is supported by the studies conducted in Benishangul-Gumuz [8], Zimbabwe [21], Saudi Arabia [17], Norway [27], China [28], and the United States [24]. The possible justification for this could be that when the nurses have many years of experience, their exposure to clinical practice becomes long, which might allow them to communicate with their colleagues, share information, and acquire knowledge regarding nonpharmacological pain management.

Working unit is the other facility-related variable significantly associated with nurses' knowledge. Nurses who work in orthopedics, surgical, emergency, and ICU were nearly six, five, five, and three times more likely to have adequate knowledge, respectively, as compared with nurses who work in pediatric units. This finding is supported by a study conducted in Turkey [26]. The possible justification for this finding could be that nurses working in orthopedic wards, surgical wards, and emergency wards might be more exposed to pain as compared to nurses working in pediatric units. Furthermore, according to statistics in Ethiopia, most commonly patients who are admitted to the orthopedic and surgical wards are adults, so those patients are more capable of complaining their feelings of pain to nurses verbally at any time as compared to pediatric patients. These might enable nurses to communicate with their colleagues and physicians regarding different modalities of pain management. It also enables them to read recent journals to update their knowledge.

In this finding, nurse-to-patient ratio was significantly associated with nurses' knowledge. Those nurses who reported a nurse-to-patient ratio of 1 : 4 are more than two times more likely to have adequate knowledge as compared to those nurses who reported an undetermined nurse-to-patient ratio. This finding is supported by studies conducted in Benishangul-Gumuz [8] and Uganda [29]. The possible reason for this could be that when there is a higher nurse-to-patient ratio, nurses might have a workload and they might not get enough time to update their knowledge, and nurse-to-patient interaction will become very limited, which makes nurses underestimate the patient's pain.

Working hours is another significantly associated factor with knowledge. Those nurses working eight hours per day were two times more likely to have adequate knowledge as compared with nurses who work more than 12 hours per day. This finding is supported by a study conducted in Saudi Arabia [17]. The possible justification could be that while nurses work more than 12 hours per day, they could become more tired and loaded. As a result, they might have no adequate time to update their knowledge.

Regarding factors associated with attitude, this study revealed that monthly income, nurse-to-patient ratio, and knowledge were significantly associated. Those nurses who have a monthly income of >8017 Ethiopian birr, 7071–8017 Ethiopian birr, and 6193–7071 Ethiopian birr were four times, five times, and three times more likely to have a favorable attitude, respectively, compared with nurses having a monthly income of less than 5358 ETB. The reason for this relationship could be because most of the time, nurses who have a relatively higher monthly income are those who have higher educational qualifications and many years of experience in nursing practice. That means these nurses could get adequate knowledge regarding nonpharmacological pain management, and they could have a favorable attitude. Moreover, nurses who have higher monthly income may afford to buy electronic products like laptop computers and smartphones, so they might access available professional-related information at their fingertips and update their knowledge.

Those nurses who reported a nurse-to-patient ratio of 1 : 4 are about two times more likely to have a favorable attitude as compared to nurses who reported an undetermined nurse-to-patient ratio. The finding of this study is supported by the studies conducted in Benishangul-Gumuz and Australia [8, 30]. The possible reason for this could be that as the nurse-to-patient ratio increases, nurses become challenged by workload and shortage of time, and this could not enable them to read more about nonpharmacological pain management, and their attitude could be changed.

Knowledge was another factor significantly associated with attitude. Those nurses who have adequate knowledge were four times more likely to have a favorable attitude towards nonpharmacological pain management as compared to nurses who have inadequate knowledge. This finding is supported by the studies conducted in Benishangul-Gumuz [8], Saudi Arabia [17], and Turkey [26]. This could be because nurses who have adequate knowledge might have enough information regarding the benefits of nonpharmacological pain management over pharmacological pain management, and this could lead nurses to have a favorable attitude.

## 5. Limitations of the Study

In this study, qualitative data collection methods were not used. The results of the study might be affected by information bias. Responses might not accurately show the attitude of nurses because the questionnaire has a self-reporting nature.

## 6. Conclusion

More than half and nearly half of the nurses had adequate knowledge and a favorable attitude towards nonpharmacological pain management, respectively. Educational qualification, years of experience, working unit, nurse-to-patient ratio, and working hours per day were significantly associated with a nurse's knowledge. Monthly income, nurse-to-patient ratio, and nurse's knowledge were significantly associated with the attitudes of nurses.

## Figures and Tables

**Table 1:** Sociodemographic characteristics of nurses working at Amhara region Comprehensive Specialized Hospitals, Ethiopia, in 2021 (*N* = 775).

Variables	Category	Frequency	Percent
*Age*	21–29 years	327	42.2
*M* = 30	30–39 years	346	44.6
IQR = 28–35	40–49 years	94	12.1
*R* = 23–57	>50 years	8	1

*Sex*	Male	332	42.8
	Female	443	57.2

*Religion*	Orthodox	507	65.4
	Protestant	78	10.1
	Muslim	163	21
	Others	27	3.5

*Marital status*	Single	299	38.6
	Married	429	55.4
	Divorced	33	4.3
	Windowed	14	1.8

*Monthly income*	<5358 ETB	341	44
*M* = 6000	5358–6193 ETB	110	14.2
IQR = 2100	6193–7071 ETB	151	19.5
*R* = 3600–11600	7071–8017 ETB	113	14.6
	>8017 ETB	60	7.74

ETB = Ethiopian birr; *M* = mean; SD = standard division; *R* = range.

**Table 2:** Professional and organizational related factors among nurses working at Amhara region Comprehensive Specialized Hospitals, Ethiopia, in 2021 (*N* = 775).

Variables	Category	Frequency	Percent
Nurse-to-patient ratio	1 : 4	236	30.5
	1 : 6	99	12.8
	1 : 8	141	18.2
	Undetermined	299	38.6

Presence of workload	Yes	546	70.5
	No	229	29.5

Is there NPPM equipment?	Yes	319	41.2
	No	456	58.8

NPPM guideline	Yes	516	66.6
	No	259	33.4

Lack of evidence	Yes	400	51.6
	No	375	48.4

Working hours	8 hours	431	55.6
	9–12 hours	216	27.9
	>12 hours	128	16.5

Pain assessment tool	Yes	380	49
	No	395	51

Do you have training on NPPM?	Yes	189	24.4
	No	586	75.6

Previous education on NPPM?	Yes	415	53.5
	No	360	46.5

Have you ever used NPPM?	Yes	608	78.5
	No	167	21.5

Educational qualification	Diploma	225	29
	BSc	462	59.6
	MSc	88	11.4

Years of experience	<1 year	140	18.1
	1–3 years	144	18.6
	3-5 years	133	17.2
	>5 years	358	46.2

Working unit	Pediatrics	146	18.8
	Oncology	14	1.8
	OPD	151	19.5
	Emergency	90	11.6
	OR	76	9.8
	Orthopedics	38	4.9
	Recovery	32	4.1
	ICU	58	7.5
	Medical ward	117	15.1
	Surgical ward	53	6.8

**Table 3:** Knowledge of nurses regarding nonpharmacological pain management among nurses working at Amhara region Comprehensive Specialized Hospitals, Ethiopia, in 2021 (*n* = 775).

Statements	Yes		No	
	*N*	*P*	*N*	*P*
The most accurate judge of the intensity of the patient's pain is the patient himself	663	85.5	112	14.5
Providing a suitable room temperature and good air conditioning can alleviate pain	680	87.7	95	12.3
Providing the patient with a possibility to rest by minimizing noise can alleviate pain	666	85.9	109	14.1
Involving families in pain management can increase a patient's ability to manage pain	617	79.6	158	20.4
Using nonpharmacological pain management has no value to the patient	168	21.7	607	78.3
Encouraging patients to relax different parts of their bodies alleviates the sensation of pain	670	86.5	105	13.5
Try to focus a patient's thoughts/attention away from pain can decrease pain	639	82.5	136	17.5
Vital signs are always reliable indicators of the intensity of a patient's pain	362	46.7	413	53.3
Asking patients to suggest ways to relieve pain can increase the patient's ability to manage pain	639	82.5	136	17.5
Patients who can be distracted from pain usually do not have severe pain	302	39	473	61
Nonpharmacological interventions are effective only for mild pain control	495	63.9	280	36.1
The patient's pain can be alleviated by position changes	626	80.8	149	19.2
The benefit of nonpharmacological pain management is that it has fewer side effects than drugs	293	37.8	482	62.2
Patients who can be distracted from pain usually do not have pain	222	28.6	553	71.4
Distraction, for example, by the use of music or relaxation, can decrease the perception of pain	650	83.9	125	16.1
Patients with chronic pain should receive pain medications along with nonpharmacological interventions at regular intervals with or without the presence of discomfort	540	69.7	235	30.3
The patient should be advised to use nonpharmacological means alone rather than medications	382	49.3	393	50.7
Nonpharmacological methods of pain relief have no applications for neonates	308	39.7	467	60.3
Nonpharmacological pain management only includes massage, heat/cold and relaxation	319	41.2	456	58.8
It may often be useful to give a placebo to assess whether a patient in pain is genuinely in pain	396	51.1	379	48.9

*N* = frequency; *P* = percent.

**Table 4:** Attitude of nurses towards nonpharmacological pain management among nurses working at Amhara region Comprehensive Specialized Hospitals, Ethiopia, in 2021 (*N* = 775).

Question	SD	D	N	A	SA
Pain cannot be seen in the patient's behavior	346	175	92	101	61
Nonpharmacological therapies should be given to sick people	71	116	119	203	266
Distraction increases the intensity of pain	288	156	131	150	50
Nondrug interventions are very effective for severe pain than mild to moderate pain	291	199	112	114	59
Using pain assessment tools usually makes nursing more complicated and consumes time for other ward activities	228	190	139	135	83
Nurses are best judges of the patient's pain intensity than doctors because they spend 24 hours with the patient	65	84	85	162	379
Nonpharmacological pain management education received during nurse training is adequate for effective pain management after graduation	238	182	128	142	85
The nurse's role during nondrug pain management is to follow only the doctor's orders	343	193	100	94	45
Are you willing to provide information related to nondrug methods to patients?	51	93	126	255	250
Are you willing to provide nonpharmacological methods to people who have pain?	50	86	118	253	268
It is not advised to use both pharmacological and nonpharmacological methods together	356	152	89	108	70
Preparing a patient for a procedure by explaining the procedure can decrease pain	68	110	102	225	270
Encouraging a patient in pain to think about pleasant and positive matters can relieve pain	47	84	119	262	263
Teaching patients about the correct breathing technique cannot alleviate his/her pain	263	188	130	133	61
Encouraging the patient by rewarding verbally cannot alleviate his/her pain	242	218	144	124	47
Encouraging the patient to relax different parts of his body can alleviate pain	42	95	104	246	288
Internal decoration of units does not affect the patient's ability to manage pain	225	255	122	105	68
Are you willing to encourage family members to bring some of the patient's belongings (pictures, pillows, etc.) to the unit?	56	78	116	225	300

SD = strongly disagree; D = disagree; N=neutral; A = agree; SA = strongly agree.

**Table 5:** Factors associated with nurses' knowledge regarding nonpharmacological pain management among nurses working at Amhara region Comprehensive Specialized Hospitals, Ethiopia, in 2021 (*N* = 775).

Variables	Category	Knowledge		COR (95% CI)	AOR (95% CI)	*p* value
		Adequate	Inadequate			
Age (years)	<31	208	247	1	1	
	>31	212	108	2.33 (1.73, 3.13)	1.42 (0.95, 2.14)	0.084

Sex	Male	118	144	1.18 (0.89, 1.58)	1.37 (0.95, 1.97)	0.088
	Female	232	211	1	1	

Monthly income (ETB)	<5358	123	219	1	1	
	5358–6193	64	46	2.47 (1.59 3.84)	0.78 (0.41, 1.45)	0.433
	6193–7071	100	51	3.49 (2.33, 5.22)	0.57 (0.29, 1.13)	0.112
	7071–8017	85	28	5.40 (3.34, 8.74)	1.85 (0.95, 3.58)	0.068
	>8017	48	11	7.76 (3.89, 15.51)	1.08 (0.39, 2.99)	0.870

Educational qualification	Diploma	63	162	1	1	
	BSc	284	178	4.10 (2.92, 5.79)	2.86 (1.80, 4.56)^*∗*^	<0.001
	MSc	73	15	12.51 (6.68, 23.43)	3.51 (1.37, 8.99)^*∗*^	0.009

Years of experience	<1 year	35	105	1	1	
	1–3 years	57	87	1.96 (1.18, 3.26)	2.19 (1.20, 4.00)^*∗*^	0.010
	3–5 years	76	57	4.00 (2.39, 6.68)	3.99 (2.05, 7.79)^*∗*^	<0.001
	>5 years	252	106	7.13 (4.57, 11.12)	5.59 (2.86, 10.94)^*∗*^	<0.001

Education on NPPM	Yes	263	194	1.39 (1.04, 1.85)	1.11 (0.77, 1.59)	0.564
	No	157	161	1	1	

Need to have education on NPPM	Yes	321	257	1.23 (0.89, 1.70)	1.31 (0.86, 2.02)	0.205
	No	99	98	1	1	

Working unit	Pediatrics	61	85	1	1	
	Oncology	8	6	1.86 (0.80, 7.85)	2.08 (0.50, 8.71)	0.312
	OPD	64	87	1.02 (0.64, 1.62)	1.41 (0.80, 2.47)	0.227
	Emergency	63	27	3.25 (1.86, 5.68)	5.10 (2.57, 10.11)^*∗*^	<0.001
	OR	38	38	1.39 (0.79, 2.43)	1.18 (0.61, 2.29)	0.614
	Orthopedic	26	12	3.01 (1.41, 6.44)	5.61 (2.25, 13.96)^*∗*^	<0.001
	Recovery	23	9	3.56 (1.54, 8.23)	2.05 (0.76, 5.57)	0.156
	ICU	43	15	3.99 (2.03, 7.83)	3.06 (1.38, 6.77)	0.006
	Medical	55	62	1.23 (0.75, 2.01)	1.41 (0.77, 2.58)	0.252
	Surgical	38	15	3.53 (1.78, 6.98)	5.29 (2.32, 12.05)^*∗*^	<0.001

Nurse-to-patient ratio	1 : 4	175	61	3.17 (2.19, 4.58)	2.33 (1.44, 3.78)^*∗*^	0.001
	1 : 6	52	47	1.22 (0.77, 1.92)	1.05 (0.60, 1.84)	0.857
	1 : 8	51	90	0.62 (0.41, 0.94)	0.38 (0.22, 0.64)^*∗*^	<0.001
	Undetermined	142	157	1	1	

Presence of workload	Yes	248	276	0.41 (0.30, 0.56)	0.63 (0.42, 0.972)	0.036
	No	172	79	1	1	

Lack of evidence	Yes	186	214	0.52 (0.39, 0.69)	0.835 (0.57, 1.20)	0.335
	No	234	141	1	1	

Working hours per day	8 hours	252	179	2.19 (1.46, 3.28)	2.15 (1.27, 3.62)^*∗*^	0.004
	8–12 hours	118	98	1.87 (1.20, 2.93)	2.09 (1.18, 3.70)^*∗*^	0.011
	>12 hours	50	78	1	1	

Ever used NPPM?	Yes	346	275	1.36 (0.95, 1.93)	1.24 (0.79, 1.93)	0.336
	No	74	80	1	1	

On-the-job training?	Yes	132	88	1.391 (1.01, 1.90)	0.98 (0.64, 1.49)	0.945
	No	288	267	1	1	

*Note* . Statistical significance: ^*∗*^*p* value <0.05.

**Table 6:** Factors associated with nurses' attitude towards nonpharmacological pain management among nurses working at Amhara region Comprehensive Specialized Hospitals, Ethiopia, in 2021 (*N* = 775).

Variables	Category	Attitude		COR (95% CI)	AOR (95% CI)	*p* value
		Favorable	Unfavorable			
Age (years)	<31	200	255	1	1	
	>31	186	134	1.77 (1.32, 2.36)	1.24 (0.84, 1.84)	0.266

Monthly income (ETB)	<5358	111	231	1	1	
	5358–6193	55	55	2.08 (1.34, 3.22)	1.26 (0.68, 2.33)	0.455
	6193–7071	93	58	3.33 (2.24, 4.97)	3.19 (1.65, 6.16)^*∗*^	0.001
	7071–8017	82	31	5.50 (3.43, 8.81)	5.36 (2.78, 10.33)^*∗*^	<0.001
	>8017 ETB	45	14	6.68 (3.52,12.70)	4.38 (1.64, 11.69)^*∗*^	0.003

Educational qualification	Diploma	75	150	1	1	
	BSc	243	219	2.21 (1.59, 3.09)	0.82 (0.52, 1.29)	0.395
	MSc	68	20	6.80 (3.84,12.02)	0.83 (0.33, 2.04)	0.687

Years of experience	<1 year	42	98	1	1	
	1–3 years	55	89	1.44 (0.88, 2.36)	0.99 (0.55, 1.77)	0.989
	3–5 years	77	56	3.20 (1.94, 5.28)	1.84 (0.96, 3.52)	0.062
	>5 years	212	146	3.38 (2.23, 5.14)	0.72 (0.38, 1.36)	0.321

Education on NPPM	Yes	222	193	1.37 (1.03, 1.82)	1.25 (0.88, 1.76)	0.204
	No	164	196	1	1	

Need to have NPPM education	Yes	298	280	1.31 (0.95, 1.82)	1.38 (0.92,2.07)	0.119
	No	88	109	1	1	

Working unit	Pediatrics	67	79	1	1	
	Oncology	8	6	1.57 (0.88, 9.83)	2.22 (0.52, 9.37)	0.275
	OPD	74	77	1.13 (0.71, 1.78)	1.35 (0.78, 2.34)	0.280
	Emergency	42	48	1.03 (0.60, 1.74)	0.67 (0.35, 1.29)	0.241
	OR	34	42	0.95 (0.54, 1.66)	0.72 (0.37, 1.40)	0.334
	Orthopedic	23	15	1.80 (0.87, 3.74)	1.89 (0.78, 4.59)	0.158
	Recovery	19	13	1.72 (0.79, 3.59)	0.80 (0.32, 2.01)	0.649
	ICU	36	22	1.92 (1.03, 1.64)	0.97 (0.46, 2.05)	0.954
	Medical	54	63	1.01 (0.62, 2.29)	1.01 (0.55, 1.84)	0.970
	Surgical	27	26	1.22 (0.65, 2.29)	0.87 (0.41, 1.86)	0.736

Nurse-to-patient ratio	1 : 4	165	71	2.90 (2.02, 4.15)	1.89 (1.19, 3.01)^*∗*^	0.007
	1 : 6	45	54	1.04 (0.65, 1.64)	0.96 (0.56, 1.65)	0.893
	1 : 8	43	98	0.54 (0.35,0.83)	0.56 (0.34, 0.94)	0.029
	Undetermined	133	166	1	1	

Work load	Yes	239	307	0.43 (0.31, 0.59)	0.70 (0.47, 1.06)	0.097
	No	147	82	1	1	

Lack of evidence	Yes	179	221	0.65 (0.49, 0.87)	0.99 (0.69, 1.42)	0.979
	No	207	168	1	1	

Working hours	8 hours	227	204	1.52 (1.02, 2.27)	0.88 (0.53, 1.44)	0.617
	8–12 hours	105	111	1.29 (0.83, 2.01)	0.86 (0.50, 1.47)	0.591
	>12 hours	54	74	1	1	

Knowledge	Adequate	291	129	6.17 (4.51, 8.44)	4.26 (2.91, 6.24)^*∗*^	<0.001
	Inadequate	95	260	1	1	

*Note*. Statistical significance: ^*∗*^*p* value <0.05.

## Data Availability

All data are available from the corresponding author upon reasonable request.

## Abbreviations

AOR: Adjusted odd ratio
CI: Confidence interval
DBCSH: Debreberhan Comprehensive Specialized Hospital
DMCSH: Debre Tabor Comprehensive Specialized Hospital
DCSH: Dessie Comprehensive Specialized Hospital
DMCSH: Debre Markos Comprehensive Specialized Hospital
ETB: Ethiopian birr
FHCSH: Felege-Hiwot Comprehensive Specialized Hospital
GUCSH: Gondar University Comprehensive Specialized Hospital
IQR: Interquartile range
NPPM: Nonpharmacological pain management
OR: Odds ratio
TGCSH: Tibebe-Ghion Comprehensive Specialized Hospital.
